# Real-world study of patients with locally advanced HNSCC in the community oncology setting

**DOI:** 10.3389/fonc.2023.1155893

**Published:** 2023-08-17

**Authors:** Christopher M. Black, Karthik Ramakrishnan, Eric Nadler, Wan-Yu Tseng, Chuck Wentworth, John Murphy, Nicole Fulcher, Liya Wang, Melannie Alexander, Gregory Patton

**Affiliations:** ^1^ Center for Observational and Real-World Evidence, Merck & Co., Inc., Rahway, NJ, United States; ^2^ Texas Oncology, Medical Oncology, Dallas, TX, United States; ^3^ Real World Research, Ontada, Boston, MA, United States; ^4^ Epidemiology, Merck & Co., Inc., Rahway, NJ, United States

**Keywords:** head and neck cancer, locally advanced, outcomes, HPV, real world, cancer treatment patterns, concurrent chemotherapy with radiation therapy, CRT

## Abstract

**Introduction:**

There is a need to understand the current treatment landscape for LA HNSCC in the real-world setting.

**Methods:**

This retrospective study assessed real-world outcomes and treatment patterns of 1,158 adult patients diagnosed with locally advanced (stage III-IVB) HNSCC initiating chemoradiotherapy (CRT) within the period January 2015 to December 2017 in a large network of US community oncology practices. Structured data were abstracted from electronic health records. Demographic, clinical and treatment characteristics were analyzed descriptively overall and stratified by index treatment (cisplatin + radiotherapy [RT], cisplatin + other chemotherapy + RT, or cetuximab + RT). Time to next treatment (TTNT) and overall survival (OS) were measured using the Kaplan-Meier method, and median duration of treatment was assessed. OS was compared across treatment cohorts using multinomial logistic regression with inverse probability treatment weighting. To identify covariates associated with OS, a multivariable adjusted Cox proportional hazard model was used.

**Results:**

This study examined 22,782 records, of which 2124 had stage III to stage IVB and no other cancers, and 1158 met all eligibility criteria. Among the treatment cohorts analyzed (cisplatin + RT, cisplatin + other chemotherapy + RT, or cetuximab + RT), cisplatin + RT was the most common concurrent chemotherapy (65.8%). Among 1158 patients, 838 (72.4%) did not initiate subsequent treatment and 139 (12.0%) died. The median TTNT and median OS were only reached by the cetuximab + RT cohort. Among patients with oropharynx primary tumor location, patients with human papilloma virus (HPV) positive status had the longest time on treatment and highest survival at 60 months. Covariates associated with improved survival were never/former tobacco use, HPV positive status, and overweight or obese body mass index. Covariates associated with poorer survival were age of 60+ years, primary tumor location of hypopharynx or oral cavity and Eastern Cooperative Oncology Group performance status score of 2+.

**Conclusion:**

These data describe real-world treatment patterns in locally advanced head and neck squamous cell cancer and sets the baseline to assess outcomes for future studies on the community oncology population.

## 1 Introduction

Head and neck cancers are malignant neoplasms occurring in the oropharynx, larynx, oral cavity, and hypopharynx regions, as well as other parts of the head and neck, and 90% of these are squamous cell cancers (SCC) (1, 2). Most head and neck cancers arise in the epithelial lining of the oral cavity, oropharynx, larynx and hypopharynx. In the United States, head and neck cancers account for nearly 3% of all cancers, with 54,540 estimated new cases diagnosed and 11,580 estimated deaths in 2023 (3). Risk factors associated with poor survival in head and neck squamous cell cancer (HNSCC) include alcohol and tobacco consumption (1), while positivity for human papillomavirus (HPV) infection is associated with better outcomes (4).

Treatment of HNSCC is guided by patient clinical characteristics and fitness for surgery. Surgery, radiotherapy, and systemic therapies including chemotherapy, targeted therapy, and immunotherapy are the most commonly used treatments for disease management in HNSCC (5). For patients with locally advanced (LA) disease or for those for whom surgical resection is not the best approach, concurrent chemotherapy with radiation therapy (CRT) is the standard of care, with improved survival outcomes compared to other treatment options (5).

Concurrent cisplatin with radiotherapy (RT) is the standard of care and has been associated with overall survival (OS) benefit (5–7). To reduce toxicity, the cisplatin is administered at a low weekly dose (5). For patients with locally advanced HNSCC for whom platinum-based chemotherapy is not suitable, cetuximab (an epidermal growth factor receptor antagonist) concurrent with radiation is associated with improved survival compared to radiation alone (8, 9).

This study ascertained patient profiles, clinical characteristics, treatment patterns, and clinical outcomes among patients with newly diagnosed LA HNSCC receiving CRT at community-based oncology practices.

## 2 Methods

This retrospective observational cohort study examined patient profiles, treatment patterns and clinical outcomes among patients with newly diagnosed LA HNSCC who initiated CRT in The US Oncology Network between 01 January 2015 and 31 December 2017. Patients were followed longitudinally until the last patient record, the end of study period (31 December 2021), or the latest available data.

### 2.1 Data source

We utilized iKnowMed™ (iKM) electronic health record (EHR) data maintained by Ontada. iKM is an oncology specific EHR system implemented across The US Oncology Network and select non-network community oncology practices. The US Oncology Network includes 1,400 affiliated physicians operating in over 500 sites of care across the United States and treats approximately 1.2 million cancer patients annually (10).

Study data were primarily sourced from the structured fields of iKM EHR data, with supplemental vital status provided by the Social Security Administration’s Limited Access Death Master File (LADMF). The iKM database captures outpatient encounter histories for patients under community-based care, including (but not limited to) patient demographics (age, race, gender, smoking status), clinical information (disease diagnosis, diagnosis stages, performance status information and laboratory test results), and cancer-directed treatment information (medication, dose, line of therapy, and start and end dates). The LADMF includes records of death reported by family members, funeral homes, hospitals, financial institutions, postal authorities, and federal agencies for persons issued a Social Security card. If there was a conflict between iKM and LADMF vital status data, LADMF data were prioritized because they contain official records of death and should be the most accurate.

### 2.2 Inclusion and exclusion criteria

The study investigated patients who were diagnosed with locally advanced (stage III – IVB, based on the AJCC 7^th^ edition) head and neck cancer with histology subtype of squamous cell carcinoma. Study data were extracted from EHRs (i.e., real-world research). This retrospective cohort study included patients aged ≥18 years with *de novo* newly diagnosed stage III-IVB HNSCC who also initiated CRT; diagnosis and treatment initiation had to be between 01 January 2015 and 31 December 2017 (study period). All eligible patients had a minimum of 2 physician visits during the study period within The US Oncology Network and had data accessible for research purposes. Patients were excluded if enrolled in interventional clinical trials, received treatment indicated for another primary cancer between 01 January 2015 and 31 December 2021, or had salivary gland cancer or nasopharynx cancer (i.e., cancer of nasopharynx, paranasal sinus, or nasal cavity). Given that the study data came from multiple choice/fill-in questions analyzed by a computer, rather than manual analysis of charts (chart review or “unstructured” data), these data are typically referred to as “structured” data in real-world research.

### 2.3 Study outcomes and cohorts

OS, treatment duration, and time to next treatment (TTNT) were assessed. Index date was defined as the first initiation date of CRT received at initial LA HNSCC diagnosis. Surgery was not considered in the TTNT. The iKM EHR structured database contains discrete fields for regimen names, drug names and visit dates. Using the regimen name field, we were able to abstract regimens containing anti-cancer drugs with radiotherapy. Index date was defined as the first initiation date of chemoradiation therapy (CRT) received at initial LA HNSCC diagnosis. TTNT was defined as the interval between index date and initiation of the next treatment or date of death due to any cause. OS was defined as the interval between the index date and the date of death (any cause) as documented in the LADMF or EHR.

Study outcomes were summarized for the overall LA HNSCC population as well as three treatment cohort types: index treatments, primary tumor locations, and HPV status. Index treatment was defined as the first CRT initiated on the index date. Index treatment cohorts included cisplatin + RT, cisplatin + other chemotherapy + RT, and cetuximab + RT. Any systemic anti-cancer drugs other than cisplatin and cetuximab indicated for LA HNSCC were considered as other chemotherapy. Primary tumor location cohorts included cancer of the hypopharynx, larynx, oropharynx, and oral cavity. HPV status included positive, negative, and not documented.

### 2.4 Statistical analysis

Descriptive methods were used to assess patient demographic, clinical, and treatment characteristics. TTNT and OS were assessed using the Kaplan-Meier method with 95% confidence intervals (CI). In TTNT, patients who did not receive a subsequent treatment and were still alive at the end of the study observation period were censored on the end of the study observation period or the last contact date available, whichever occurred first. In OS, patients who did not have a date of death documented within the study observation period were censored on the end of the study observation period or the last contact date available, whichever came first.

Independent associations of all baseline variables with OS were conducted using a Cox proportional hazard model. All baseline demographic and clinical variables, including age at diagnosis, gender, race, region, stage at diagnosis, primary tumor location, smoking history, body mass index (BMI), Eastern Cooperative Oncology Group performance status (ECOG PS) score, HPV status and index treatments, were included as independent variates in a univariate model. The hazard ratio (HR) and its CI were estimated with ties handled by Breslow (11). For the Cox proportional hazard model building, a type 3 p-value for entry of 0.20 and a type 3 p-value for retention of 0.10 were set a priori. Stepwise selection and clinically relevant covariates were used to build the model.

Cox proportional hazard regression with inverse probability treatment weighting (IPTW) (12), a method to account for differences in cohorts, was used to adjust OS results, given that elderly patients and those with poorer health are more likely to receive cetuximab (13). Multinomial logistic regression was performed to estimate the probability of receiving the treatment regimens of interest, and IPTW was used to reweight and balance cohorts. Extreme values were checked via weight truncation/trimming or weight stabilization if needed. The weights were then used in a Cox regression model to estimate OS. To estimate the variance and CIs of the OS estimates, a robust sandwich variance estimator was used. While no formal cutoff was used for standardized mean difference (SMD) for IPTW analysis (12), the means of values for SMD in IPTW, as well as some general patterns about SMD, were reported.

## 3 Results

### 3.1 Study population

**Figure 1:**
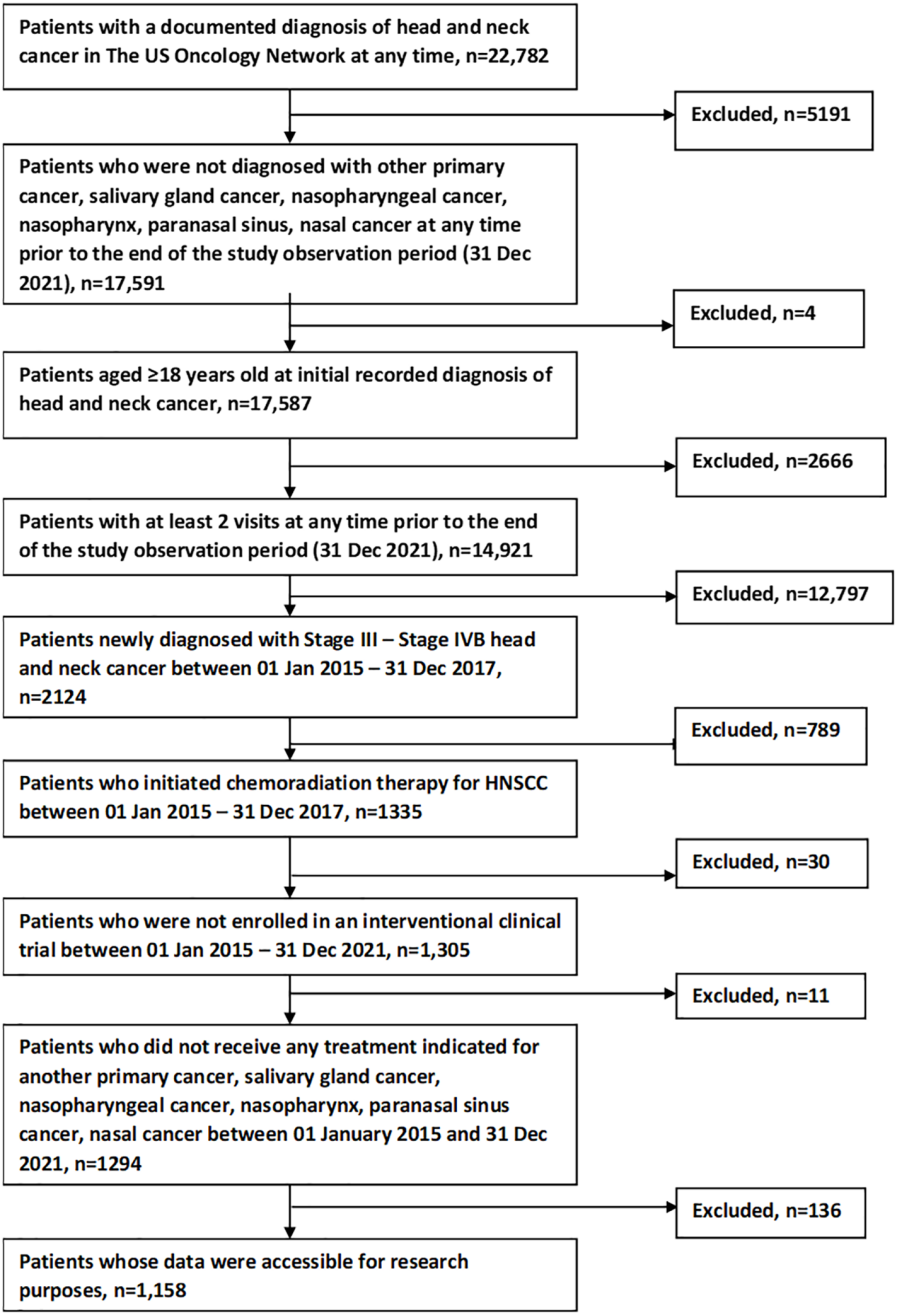
Study population attrition flow chart.

**Figure 1** shows the study population attrition. Initially, 22,782 patients were identified from The US Oncology Network with a documented diagnosis at any time of head and neck cancer. After applying inclusion and exclusion criteria, 1158 patients remained for analysis.

### 3.2 Demographics and clinical characteristics

We examined demographics (**Table 1**) and clinical characteristics (**Table 2**) at initial diagnosis for the overall study population and stratified by index treatment. The index treatments observed were cisplatin + RT (n=762, 65.8%), cisplatin + other chemotherapy + RT (n=232, 20.0%) and cetuximab + RT (n=164, 14.2%).

**Table 1:** Baseline demographic characteristics by overall and by index treatments.

Variable	Overall	Cisplatin + RT	Cisplatin + other chemo + RT	Cetuximab + RT
	n=1158	n=762	n=232	n=164
Age at diagnosis, years				
Median (min, max)	61.8 (22.8, 92.5)	60.8 (27.7, 88.0)	62.2 (22.8, 92.5)	66.6 (24.1, 90.7)
18 - 50	137 (11.8)	95 (12.5)	29 (12.5)	13 (8.0)
51- 60	406 (35.1)	297 (39.0)	80 (34.5)	29 (17.7)
61 -70	422 (36.4)	266 (34.9)	90 (38.8)	66 (40.2)
71 +	193 (16.7)	104 (13.6)	33 (14.2)	56 (34.1)
Gender, n (%)				
Male	952 (82.2)	622 (81.6)	192 (82.8)	138 (84.2)
Female	206 (17.8)	140 (18.4)	40 (17.2)	26 (15.9)
Race, n (%)				
Caucasian	947 (91.2)	634 (92.6)	185 (88.1)	128 (89.5)
African American	71 (6.8)	37 (5.4)	21 (10.0)	13 (9.1)
Asian	14 (1.4)	10 (1.5)	<5	<5
Other^a^	6 (0.6)	<5	<5	<5
Not documented	120	77	22	21
Practice location, n (%)				
Midwest	166 (14.3)	130 (17.1)	14 (6.0)	22 (13.4)
Northeast	120 (10.4)	92 (12.1)	15 (6.5)	13 (7.9)
South	366 (31.6)	185 (24.3)	125 (53.9)	56 (34.2)
West	506 (43.7)	355 (46.6)	78 (33.6)	73 (44.5)

a Other Race consists of American Indian, Native American, Native Hawaiian.

**Table 2:** Baseline clinical characteristics for overall and by index treatments.

Variable	Overall	Cisplatin + RT	Cisplatin + other chemo + RT	Cetuximab + RT
	n=1158	n=762	n=232	n=164
BMI at baseline				
Patients with available data	1128	748	224	156
Median (Min, Max)	26.7 (14.9, 48.2)	26.8 (15, 45.5)	27.3 (15.6,47.3)	25.5 (14.9, 48.2)
BMI at baseline (categorical), n (%)				
Underweight (< 18.5 kg/m^2^)	60 (5.3)	38 (5.1)	12 (5.4)	10 (6.4)
Normal (18.5-24.9 kg/m^2^)	358 (31.7)	229 (30.6)	70 (31.3)	59 (37.8)
Overweight (25.0-29.9 kg/m^2^)	400 (35.5)	282 (37.7)	67 (29.9)	51 (32.7)
Obese (≥ 30 kg/m^2^)	310 (27.5)	199 (26.6)	75 (33.5)	36 (23.1)
Not documented	30	14	8	8
Tobacco use, n (%)				
Patients with available data	1099	723	218	158
No history of tobacco use	316 (28.8)	179 (24.8)	62 (28.4)	35 (22.2)
Current tobacco use	253 (23.0)	325 (45.0)	38 (17.4)	36 (22.8)
Former tobacco use	530 (48.2)	219 (30.3)	118 (54.1)	87 (55.1)
Not documented	59	39	14	6
Stage at diagnosis, n (%) (based on AJCC 7th edition), n (%)				
Patients with available data	1158			
Stage III	289 (25.0)	190 (24.9)	49 (21.1)	50 (30.5)
Stage IV	12 (1.0)	<5	6 (2.6)	<5
Stage IVA	796 (68.7)	526 (69.0)	160 (69.0)	110 (67.1)
Stage IVB	61 (5.3)	42 (5.5)	17 (7.3)	<5
TNM staging at diagnosis, n (%)				
Primary tumor (T), n (%)				
Patients with available data	746	520	135	104
TX	30 (4.0)	23 (4.4)	<5	<5
T1	141 (18.9)	108 (20.8)	20 (14.8)	15 (14.4)
T2	301 (40.4)	208 (40.0)	56 (41.5)	43 (41.4)
T3	201 (26.9)	133 (25.6)	42 (31.1)	30 (28.9)
T4	73 (9.8)	48 (9.2)	13 (9.6)	12 (11.5)
Regional lymph nodes (N), n (%)				
Patients with available data	742	517	136	109
NX	11 (1.5)	8 (1.6)	<5	9 (8.3)
N0	70 (9.4)	50 (9.7)	11 (8.1)	11 (10.1)
N1	141 (19.0)	97 (18.8)	23 (16.9)	23 (21.1)
N2	498 (67.1)	347 (67.1)	95 (69.9)	65 (59.6)
N3	22 (3.0)	15 (2.9)	6 (4.4)	<5
Distant metastasis (M), n (%)				
Patients with available data	714	494	132	101
M0	714 (100)	494 (100)	132 (100)	101 (100)
M1	0 (0)	0 (0)	0 (0)	0 (0)
Initial tumor location(s), n (%)				
Patients with available data	1158	762	232	164
Hypopharynx	84 (7.3)	53 (7.0)	19 (8.2)	12 (7.3)
Larynx	164 (14.2)	103 (13.5)	29 (12.5)	32 (19.5)
Lip and oral cavity	135 (11.7)	92 (12.1)	33 (14.2)	10 (6.1)
Oropharynx	775 (66.9)	514 (67.5)	151 (65.1)	110 (67.1)
ECOG PS at index date, n (%)				
Patients with available data	982	684	165	133
0	241 (24.5)	189 (27.6)	31 (18.8)	21 (15.8)
1	637 (64.9)	444 (64.9)	109 (66.1)	84 (63.2)
2	100 (10.2)	51 (7.5)	22 (13.3)	27 (20.3)
≥3	<5	0	<5	<5
Not documented	176	78	67	31
HPV status among overall patients, n (%)				
Patients with available data	522	367	80	75
Positive	361 (69.2)	256 (69.8)	52 (65.0)	53 (70.7)
Negative	132 (25.3)	90 (24.5)	24 (30.0)	18 (24.0)
Tested but result undetermined	29 (5.6)	21 (5.7)	<5	<5
Not documented	636	395	152	89
HPV status among oropharynx cancer, n (%)				
Patients with available data	403	278	65	60
Positive	318 (78.9)	227 (81.7)	46 (70.8)	45 (75.0)
Negative	71 (17.6)	44 (15.8)	16 (24.6)	11 (18.3)
Tested but result undetermined	14 (3.5)	7 (2.5)	<5	<5
Not documented	372	236	21	50

AJCC, American Joint Committee on Cancer; BMI, body mass index; ECOG PS, Eastern Cooperative Oncology Group; HPV, human papilloma virus; RT, radiation therapy.

Demographic characteristics (**Table 1**) were generally numerically similar across the treatment cohorts. The cetuximab + RT cohort was numerically older (median 66.6 years), followed by cisplatin + other chemotherapy + RT (62.2 years) and cisplatin + RT (60.8 years). Most patients were male (82.2%) and Caucasian (91.2%). Most patients received treatment at practices in the West (43.7%) and South (31.6%), although the South was the most common for the cisplatin + other chemotherapy + RT cohort (53.9%).

Clinical characteristics were also numerically similar across cohorts, with notable exceptions (**Table 2**). The cetuximab + RT cohort had the lowest proportion of patients that were overweight or obese (55.8% for cetuximab + RT vs. 64.3% for cisplatin + RT vs. 63.4% for cisplatin + other chemotherapy + RT). The cisplatin + RT cohort had the highest proportion of current tobacco users (45.0%), while former tobacco use accounted for the highest proportion of the cisplatin + other chemotherapy + RT and the cetuximab + RT cohorts (54.1% and 55.1%, respectively). Oropharynx was the most common primary tumor site overall and across index treatment cohorts, found in 775 subjects overall (66.9%). The cisplatin + RT cohort had the numerically highest proportion of patients with ECOG PS score of 0-1 (92.5%), followed by cisplatin + other chemo + RT (84.9%) and cetuximab + RT (79.0%) (**Table 2**).

Stages at diagnosis, tumor site at diagnosis, and fraction HPV positive were numerically similar among treatment groups. For stages, in the overall group, 25.0% were stage III and 68.7% were stage IVA. TNM classification distribution and tumor location were similar among cohorts. HPV status was positive for the majority of patients overall (69.2%) and for those with oropharynx as the primary tumor site (78.9%). However, HPV status was not documented in the overall study population for 51.8% of the cisplatin + RT cohort, 65.5% of the cisplatin + other chemotherapy + RT cohort, and 54.3% of the cetuximab + RT cohort, and in the oropharynx population for 45.9%, 13.9%, and 45.5% of the same respective treatment cohorts.

### 3.3 Treatment patterns

**Figure 2:**
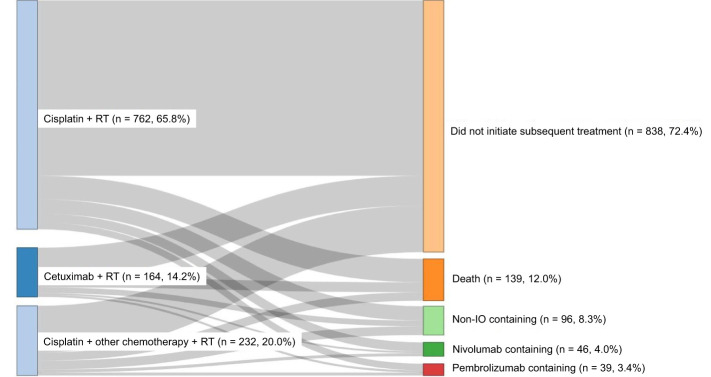
Treatment sequencing from index treatment to first subsequent treatment.

Among the treatment cohorts analyzed (cisplatin + RT, cisplatin + other chemotherapy + RT, or cetuximab + RT), cisplatin + RT was the most common concurrent CRT (65.8%) (**Figure 2**).

For the subsequent treatment (**Figure 2**) among the overall study population, 838 (72.4%) did not initiate subsequent treatment, 139 (12.0%) died, 96 (8.3%) received non-immune checkpoint inhibitor (immuno-oncology medications, IO)-containing regimens, 46 (4.0%) received nivolumab-containing regimens, and 39 (3.4%) received pembrolizumab-containing regimens.

### 3.4 Outcomes

The median follow-up duration for the overall population was 22.7 months (**Table 3A**). Cisplatin + RT had the longest median follow-up period (25.2 months), followed by cisplatin + other chemotherapy + RT (21.5 months) and cetuximab + RT (12.0 months) (**Table 3A**). The median treatment durations among these three treatment groups were numerically similar (all 1.4 months, data not shown).

**Table 3:** Overall survival values stratified by index treatment, tumor location and, for oropharynx, by HPV status. TABLE 3A Overall survival by index treatment group.

Variable	Overall n=1158	Cisplatin + RT n=762	Cisplatin + other chemo + RT n=232	Cetuximab + RT n=164
Follow-up duration (months), median (min, max)	22.7 (0.03, 83.1)	25.2 (0.03, 83.1)	21.5 (0.03, 78.9)	12.0 (0.03, 80.5)
Events, n (%)	236 (20.4)	124 (16.3)	57 (24.6)	55 (33.5)
Mean (SE), months	54.8 (0.8)	58.1 (1.0)	44.1 (1.5)	38.6 (2.2)
Median (95% CI)	NR (NR,NR)	NR (NR,NR)	NR (NR,NR)	54.9 (35.4, NR)
Q1, Q3	39.5, NR	59.7, NR	33.8, NR	14.7, NR
Survival probability, % (95% CI)				
12 months	88.0 (85.8,89.9)	91.1 (88.6,93.0)	85.7 (80.0,89.9)	76.2 (67.9,82.6)
24 months	82.0 (79.3,84.4)	86.4 (83.3,88.9)	79.0 (72.3,84.2)	64.8 (55.2,72.8)
36 months	76.6 (73.5,79.4)	81.6 (78.0,84.6)	72.6 (64.9,78.8)	57.8 (47.4,66.8)
48 months	72.1 (68.6,75.3)	77.9 (73.9,81.4)	66.3 (57.7,73.6)	51.3 (40.3,61.3)
60 months	67.2 (63.0,71.0)	74.3 (69.4,78.5)	60.4 (50.6,68.9)	42.8 (30.2,54.7)
72 months	63.2 (57.0,68.7)	68.4 (60.0,75.4)	60.4 (50.6,68.9)	42.8 (30.2,54.7)
84 months	NA	NA	NA	NA

CI, confidence interval; NA, not available; NR, not reached; OS, overall survival; RT, radiation therapy; SE, standard error.

Factors associated with OS were analyzed with multivariable (not univariable) Cox analysis. Patients with no documentation were eliminated and a total of 454 patients remained in the final multivariate model. The following covariates were associated with improved survival: never having used tobacco (HR = 0.66; p=0.04), former tobacco user (HR = 0.66, p = 0.01), HPV status of not documented (HR = 0.69 p = 0.05) or positive (HR = 0.32, p<0.0001), overweight BMI (HR = 0.60, P = 0.0039), and obese BMI (HR = 0.54, p=0.002). Covariates associated with poorer survival were age of 61-70 years (HR = 1.67, p=0.001) or 71+ years (HR = 1.8, p=0.004), primary tumor location of hypopharynx (HR = 1.87, p=0.005) or oral cavity (HR = 2.71, p<0.0001) and ECOG PS score of 2+ (HR = 3.27, p<0.0001). Additionally, the results of the univariable Cox model (**Supplementary Table 1**) are generally in agreement with this multivariable analysis.

TTNT (data not shown) was measured overall and stratified by index treatment. Median TTNT was reached only for the cetuximab + RT cohort (30.4 months). The cisplatin + RT cohort had the numerically highest likelihood of not initiating a subsequent treatment after index at 60 months, followed by the cisplatin + other chemotherapy + RT and the cetuximab + RT cohorts (65.0%, 55.2%, and 39.5%, respectively).

**Figure 3:**
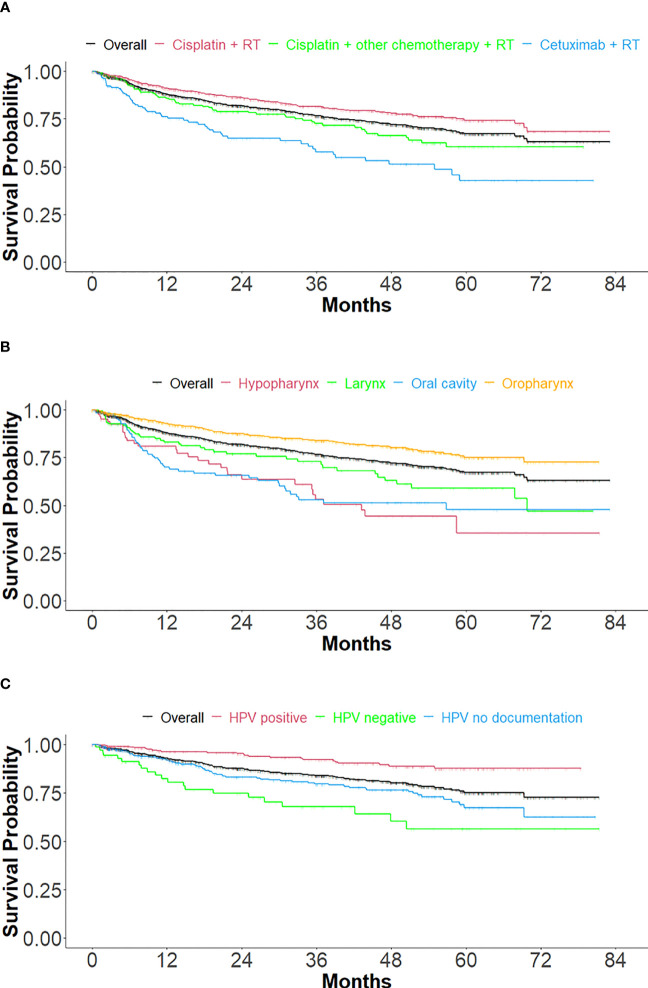
Kaplan Meier curves of overall survival, stratified by treatment, tumor location, and HPV status. **(A)** Stratified by index treatment **(B)** Stratified by primary tumor location and **(C)** For oropharynx primary tumor location, stratified by HPV status.

OS (**Figure 3** and **Table 3**) was measured overall and stratified by index treatment (**Figure 3A**), as well as tumor location (**Figure 3B**) and HPV status (**Figure 3C**). As observed with TTNT, median OS was reached only for the cetuximab + RT cohort (54.9 months). The cisplatin + RT cohort had the highest likelihood of survival after index at 60 months, followed by the cisplatin + other chemotherapy + RT and the cetuximab + RT cohorts (74.3%, 60.4%, and 42.8%, respectively).

The mean SMD was 0.141 before IPTW analysis was performed. After applying IPTW, OS was longer in the cisplatin + RT (HR: 0.46; 95% CI: 0.39-0.55) or cisplatin + other chemotherapy + RT (HR: 0.67, 95% CI: 0.58-0.79) compared to the cetuximab + RT cohort. All covariates analyzed via IPTW for these comparisons had a SMD of <0.2 and the majority had a SMD of <0.1 (mean SMD=0.043) (data not shown), indicating the cohorts were balanced (12). The HRs prior to IPTW (**Supplementary Table 1**) of 0.4 and 0.6 were similar to the results of IPTW of 0.46 and 0.67 for cisplatin + RT and cisplatin + other chemotherapy + RT, respectively, compared to cetuximab + RT.

For primary tumor location (**Figure 3B** and **Table 3B**), the median follow-up durations/OS in months were 22.7/NR, 15.3/43.2, 14.7/69.8, 13.4/56.8, and 26.8/NR for overall (n=1158), hypopharynx (n=84), larynx (n=164), oral cavity (n=135) and oropharynx (n=775) respectively. The 60-month probability of survival was 67.2%, 35.4%, 59.1%, 47.7%, and 75.1% for overall, hypopharynx, larynx, oral cavity, and oropharynx.

**Table 3B:** Overall survival stratified by primary tumor site.

Variable	Overall n=1158	Hypopharynx n=84	Larynx n=164	Oral cavity n=135	Oropharynx n=775
Follow-up duration (months), median (min, max)	22.74 (0.0, 83.1)	15.33 (0.7, 81.5)	14.72 (0.0, 80.4)	13.4 (0.2, 80.4)	26.78 (0.0, 81.4)
Events, n (%)	236 (20.4%)	31 (36.9%)	42 (25.6%)	49 (36.3%)	114 (14.7%)
Mean (SE), months	54.8 (0.8)	37.2 (2.9)	50.5 (2.5)	36.4 (2.3)	58.9 (0.9)
Median (95% CI)	NR (NR,NR)	43.2 (24.0,NR)	69.8 (48.9,NR)	56.8 (30.1,NR)	NR (NR,NR)
Q1, Q3	39.5,NR	17.0,NR	31.2,NR	10.0,NR	69.3,NR
Survival probability, % (95% CI)					
12 months	88.0 (85.8,89.9)	80.9 (69.8,88.2)	83.1 (75.6,88.5)	70.0 (60.5,77.6)	92.9 (90.6,94.6)
24 months	82.0 (79.3,84.4)	65.9 (52.5,76.3)	77.0 (68.3,83.5)	65.7 (55.9,73.8)	87.6 (84.6,90.0)
36 months	76.6 (73.5,79.4)	53.0 (38.2,65.8)	72.9 (63.3,80.3)	52.9 (42.0,62.7)	83.9 (80.5,86.7)
48 months	72.1 (68.6,75.3)	44.2 (29.2,58.2)	63.0 (51.7,72.4)	51.3 (40.3,61.3)	80.2 (76.4,83.6)
60 months	67.2 (63.0,71.0)	35.4 (17.1,54.3)	59.1 (47.2,69.2)	47.7 (35.3,59.0)	75.1 (70.0,79.4)
72 months	63.2 (57.0,68.7)	35.4 (17.1,54.3)	47.0 (29.1,63.1)	47.7 (35.3,59.0)	72.5 (65.3,78.5)
84 months	NA	NA	NA	NA	NA

CI, confidence interval; HPV, human papilloma virus; NA, not available; NR, not reached; SE, standard error.

Treatment outcomes (**Figure 3C** and **Table 3C**) were stratified by HPV status for only the oropharynx subgroup (n=775), in cohorts of HPV positive (n=318), HPV negative (n=71), and HPV undocumented (n=386). Median OS was not reached in any of the HPV cohorts. The 60-month survival rate was greatest for the HPV-positive cohort, followed by the not documented and HPV negative cohorts (87.6%, 67.4%, and 56.4%, respectively).

**Table 3C:** Overall survival among HNSCC subjects with oropharynx tumor site location, stratified by HPV status.

Variable	Oropharynx cancer overall n=775	HPV positive n=318	HPV negative n=71	HPV no documentation n=386
Follow-up duration (months), median (min, max)	26.8 (0.03, 81.4)	34.5 (1.0, 78.5)	24.9 (0.6, 81.4)	20.9 (0.03, 80.8)
Events, n (%)	114 (14.7)	24 (7.5)	21 (29.6)	69 (17.9)
Mean (SE), months	58.9 (0.9)	51.6 (0.7)	37.9 (2.5)	56.2 (1.4)
Median (95% CI), months	NR (NR,NR)	NR (NR,NR)	NR (42.2,NR)	NR (69.3,NR)
Q1, Q3, months	69.3,NR	NR,NR	19.4,NR	51.5,NR
Survival probability, % (95% CI)				
12 months	92.9 (90.6,94.6)	96.3 (93.1,98.0)	82.3 (70.2,89.8)	92.1 (88.6,94.6)
24 months	87.6 (84.6,90.0)	95.3 (91.9,97.3)	74.7 (61.4,84.0)	83.1 (78.1,87.1)
36 months	83.9 (80.5,86.7)	92.2 (87.8,95.1)	67.7 (53.3,78.6)	79.6 (74.0,84.1)
48 months	80.2 (76.4,83.6)	88.7 (83.2,92.5)	60.4 (44.0,73.4)	76.5 (70.5,81.5)
60 months	75.1 (70.0,79.4)	87.6 (81.5,91.7)	56.4 (39.3,70.4)	67.4 (59.0,74.5)
72 months	72.5 (65.3,78.5)	87.6 (81.5,91.7)	56.4 (39.3,70.4)	62.6 (49.9,73.0)
84 months	NA	NA	NA	NA

CI, confidence interval; HPV, human papilloma virus; NA, not available; NR, not reached; SE, standard error.

## 4 Discussion

This study provides real-world insights on patient profiles, treatment patterns, and clinical outcomes stratified by CRT treatment groups, primary tumor location, and HPV status for patients with LA HNSCC (stages III-IVB as defined by AJCC 7^th^ edition), treated between 2015 and 2017 within The US Oncology Network. Patients were predominantly managed with CRT employing cisplatin or cetuximab. In our findings, the most commonly reported initial treatments for LA HNSCC were cisplatin + RT, cisplatin + other chemotherapy + RT, and cetuximab + RT, in that order. Patients who received cetuximab + RT were slightly older and had numerically higher ECOG PS scores compared to the other treatment groups, in line with guidelines of cetuximab use for those who cannot tolerate cisplatin toxicity. As expected, age, BMI, ECOG PS, HPV status, primary tumor location, and index treatment groups were significant predictors for OS. OS was longest for oropharynx primaries and shortest for hypopharynx primaries, with oral cavity and larynx cancer outcomes being intermediate.

The OS and TTNT are consistent with existing retrospective studies on LA HNSCC, especially in terms of the improved outcomes with cisplatin and covariates associated with improved outcomes. Lee et al. performed a multicenter retrospective study in Korea of 445 patients with LA HNSCC receiving combined therapeutic modalities. They found a 5-year survival rate of approximately 70%, with improved prognosis associated with HPV positivity and location of oral cavity (14). Several studies in the academic research setting have shown consistent results. In a study performed at the University of Colorado and the University of New Mexico, Stokes et al. found median OS of 58.1 months for cetuximab with radiotherapy and 97.9 months for cisplatin with radiotherapy (15). In a study at the Rajiv Gandhi Cancer Institute and Research Centre, Rawat et al. found that the median OS was significantly better with a cisplatin-preloaded group than a cetuximab-preloaded group, with both treatments followed by radiotherapy (53.6 vs. 32.6 months; p = 0.044) (16). In a large study by Sun et al. at the University of Pennsylvania and the University of Utah (17), median OS was 74.4 months for cisplatin + RT and 31.1 months for cetuximab + RT. In a study at the Loma Linda University Medical Center, Jeong et al. found that the median OS was not reached for cisplatin-based CRT vs. 132 months with cetuximab for the overall population, and not reached for cisplatin-based CRT vs. 60 months with cetuximab among HPV positive patients (18).

The median OS (**Figure 3A** and **Table 3A**) was not reached for the cisplatin cohorts, which suggests a need for studies with longer follow-up. Still, the OS rates in this study have been observed elsewhere for HNSCC (19), including LA HNSCC (14). Treatment durations for all cohorts were approximately 1.4 months, which, though seemingly short, are in line with the typical duration of RT treatment with RT of 6-7 weeks.

The results of this study should be considered in the context of the strengths and limitations of the data source and study design. First, the iKM data are collected for clinical practice purposes, not research, which may have impeded the standardization of the data collection methods, instruments, and reporting practices. The clinical practice nature of data collection and our emphasis on structured data can lead to a high amount of unavailable data. Second, the iKM EHR data in this study only contained information on patient visits in The US Oncology Network community oncology practices, not hospitalizations or outside clinics, limiting the generalizability of our results. Third, clinics using iKM adhere more rigidly to evidence-based practices and may not represent community oncology clinics that treat by other methods, again limiting the generalizability of our results. Finally, disease staging referred to the AJCC 7^th^ edition and does not reflect the newer (2018) AJCC 8^th^ edition, which down staged patients with node-positive oropharynx carcinoma.

The treatment recommendations for LA HNSCC have not changed in the past approximate 10 to 20 years. Immune checkpoint inhibitors have been shown to be effective for treating patients with recurrent or metastatic HNSCC, but data are sparse regarding LA HNSCC. The Phase 3 JAVELIN Head and Neck 100 study compared PFS for avelumab + CRT followed by avelumab maintenance vs CRT alone followed by placebo maintenance (20). Neither of the trials showed improvement with avelumab except for an exploratory analysis of patients with high PD-L1 expression in JAVELIN Ovarian 100 (20). The latter results suggested a potential PFS benefit with avelumab + CRT and highlighted the need for future translational and clinical studies in this disease area.

This study identified the differences across patients based on treatments, including cisplatin + RT, cisplatin + other chemotherapy + RT, and cetuximab + RT. Compared to patients that received a cisplatin-containing regimen + RT, patients that received cetuximab + RT were older and had higher ECOG PS scores. Older and sicker patients may be unable to tolerate cisplatin (21) and would therefore be more likely to receive cetuximab + RT (13). Patients with cetuximab + RT showed lower survival probabilities when compared to cisplatin + RT with or without chemotherapy. When compared to cisplatin + RT and cisplatin + other chemotherapy + RT, cetuximab + RT had a shorter follow-up period. In addition, among these three cohorts, cetuximab + RT had a slightly higher proportion of patients that died (33.5%) compared to cisplatin + RT (16.3%) and (24.6%). In the univariable and multivariable Cox model results, cetuximab + RT was associated with worsening OS while cisplatin + RT with or without chemotherapy were associated with prolonged OS.

According to standard care for LA HNSCC, cisplatin dosing is 40 mg/m^2^ weekly or 100 mg/m^2^ every third week (22). In addition, mucositis, dysphagia, odynophagia and disease-related nutritional challenges have been reasons for treatment discontinuation (23), as well as hematologic toxicities or infection issues. Future studies may investigate the optimal cisplatin dose and the reasons for treatment discontinuation among patients with LA HNSCC receiving care in the community-based oncology practice setting.

HPV testing plays an important role in predicting HNSCC prognosis, particularly for the oropharynx subtype. We observed a trend that patients that tested HPV positive had longer OS, which is aligned with other studies. Our study results showed that approximately 50% had HPV documentation, and of those, 78.9% were HPV positive. Guideline recommendations for HPV testing were released in 2018 (24, 25), while our study identification period was between 01 Jan 2015 and 31 Dec 2017, with follow-up till 31 Dec 2021. It is possible that some clinical practices adopted HPV testing later and that we might observe a positive trend of HPV testing rate. Future studies can warrant a better understanding of the rates of HPV testing among patients with HNSCC over time. In addition, a study limitation is that we used data from structured fields within the EHR. Patient charts may serve as a better source of HPV status data for future studies.

Diagnosing and treating cancer at an early stage may help patients achieve better outcomes and improve their quality of life. We found that in this disease area, various treatment modalities are being tested. Our study results could be beneficial for understanding patient profile, treatment patterns, disease burdens and clinical outcomes. Using the most contemporary data, this study provides current insights on how patients with LA HNSCC are treated in the community-based oncology practices in the US. In addition, this study generated real-world evidence that can be leveraged for future trials or study design considerations.

In conclusion, we found that cisplatin and cetuximab are mainly used in definitive therapies, and that the outcomes and factors associated with OS in our study match those of prior studies. This study offers insights into how the results of RCTs have translated into the treatment and outcomes for patients with LA HNSCC managed within the community oncology setting.

## Data availability statement

The data analyzed in this study are subject to the following licenses/restrictions: The datasets presented in this article are not readily available because the health data used to support the findings of this study are restricted by The US Oncology Institutional Review Board in order to protect patient privacy. For this reason, data used to support the findings of this study have not been made available. Requests to access these datasets should be directed to christopher.black2@merck.com; CB.

## Ethics statement

Institutional Review Board and Compliance/Privacy approval was gained prior to initiation of the retrospective research. Since this project involved the analysis of existing data and records, study information was analyzed in such a manner that research participants could not be directly identified. Patient informed consent was not required due to the nature of the study design. Thus, exemption status and a waiver of informed consent were approved by The US Oncology, Inc. Institutional Review Board. Data were handled in compliance with HIPAA and the Health Information Technology for Economic and Clinical Health (HITECH) Act.

## Author contributions

All authors contributed substantially to the manuscript and fit ICJME authorship criteria.
