# Supplementary material for: Real-world study of patients with locally advanced HNSCC in the community oncology setting
Source: Front Oncol. 2023 Aug 17;13:1155893. doi: 10.3389/fonc.2023.1155893 (PMC10472134; doi:10.3389/fonc.2023.1155893)
Supplement: Supplementary file 1 [file Table_1.docx]

Supplementary Table 1. Cox univariable model for overall survival

|  | **Univariable** | | |
| --- | --- | --- | --- |
| **Variable (reference)** | **HR** | **95% CI** | **P value** |
| **Age (years) (51 - 60)** |  |  |  |
| 18-50 | 1.7 | 0.7-3.9 | 0.2 |
| 61-70 | 1.9 | 1.0-3.7 | 0.05 |
| 71+ | 4.7 | 2.4-9.3 | **<0.0001** |
| **Gender (Male)** |  |  |  |
| Female | 1.6 | 0.9-2.8 | 0.1 |
| **Race (white)** |  |  |  |
| African American | 1.5 | 0.9-2.3 | 0.1 |
| Asian / Other | 1.5 | 0.6-3.5 | 0.4 |
| Not Documented | 1.0 | 0.6-1.5 | 0.9 |
| **Region (South)** |  |  |  |
| Midwest | 1.0 | 0.6-1.5 | 0.9 |
| Northeast | 1.5 | 0.9-2.3 | 0.1 |
| West | 1.5 | 0.6-3.5 | 0.4 |
| **Stage (III)** |  |  |  |
| IV/IVA | 1.0 | 0.6-1.8 | 0.9 |
| IVB | 1.8 | 0.7-5.0 | 0.2 |
| **Primary Tumor location (Oropharynx)** |  |  |  |
| Hypopharynx | 4.0 | 2.0-8.3 | **0.0001** |
| Larynx | 2.5 | 1.4-4.5 | **0.002** |
| Oral cavity | 2.3 | 0.6-9.5 | 0.3 |
| **Smoking status (current)** |  |  |  |
| Former | 0.6 | 0.5-0.8 | **0.0013** |
| Never | 0.4 | 0.3-0.7 | **<0.0001** |
| Not Documented | 0.3 | 0.2-0.7 | **0.004** |
| **BMI (Normal)** |  |  |  |
| Underweight | 0.6 | 0.4-0.9 | **0.0187** |
| Overweight | 0.2 | 0.1-0.4 | **<0.0001** |
| Obese | 0.2 | 0.1-0.4 | **<0.0001** |
| Not Documented | 0.5 | 0.2-1.0 | 0.6 |
| **ECOG PS (0)** |  |  |  |
| 1 | 2.0 | 1.3-3.1 | **0.001** |
| 2+ | 5.8 | 3.6-9.6 | **<0.0001** |
| Not Documented | 2.7 | 1.6-4.3 | **<0.0001** |
| **HPV status (negative)** |  |  |  |
| Positive | 0.2 | 0.1-0.4 | **<0.0001** |
| Not Documented | 0.9 | 0.5-1.9 | 0.9 |
| **Index treatment group**  **(Cetuximab + RT)** |  |  |  |
| Cisplatin + RT | 0.4 | 0.3-0.5 | **<0.0001** |
| Cisplatin + other chemo + RT | 0.6 | 0.4-0.9 | **0.0052** |
| BMI, body mass index; CI, confidence interval; ECOG PS, Eastern Cooperative Oncology Group performance status; HPV, human papillomavirus; HR, hazard ratio; RT, radiation therapy | | | |
